# Curcumin Inhibits Joint Contracture through PTEN Demethylation and Targeting PI3K/Akt/mTOR Pathway in Myofibroblasts from Human Joint Capsule

**DOI:** 10.1155/2019/4301238

**Published:** 2019-08-14

**Authors:** Ze Zhuang, Dongjie Yu, Zheng Chen, Dezhao Liu, Guohui Yuan, Ni Yirong, Linlin Sun, Yuangao Liu, Ronghan He, Kun Wang

**Affiliations:** ^1^Departments of Joint Surgery and Orthopedic Trauma, The Third Affiliated Hospital of Sun Yat-sen University, Guangzhou 510630, Guangdong, China; ^2^Department of Urology, The First Affiliated Hospital of Jinan University, Guangzhou 510632, Guangdong, China; ^3^Departments of Anesthesiolgy, The Third Affiliated Hospital of Sun Yat-sen University, Guangzhou 510630, Guangdong, China; ^4^MOE Key Laboratory of Laser Life Science & SATCM Third Grade Laboratory of Chinese Medicine and Photonics Technology, College of Biophotonics, South China Normal University, Guangzhou 510631, Guangdong, China; ^5^Departments of Infectious Diseases, The Third Affiliated Hospital of Sun Yat-sen University, Guangzhou 510630, Guangdong, China

## Abstract

Joint contracture is increasingly regarded as a clinical problem that leads to irreversible dysfunction of the joint. It is a pathophysiological process following joint injury, which is marked by the activation of myofibroblasts. There is currently no effective treatment for the prevention of joint contracture. Curcumin is a polyphenol pigment extracted from turmeric, which possesses anti-inflammatory, antioxidative, and antitumor properties. In the present study, we demonstrated that curcumin exerts a protective effect against joint contracture via the inhibition of myofibroblast proliferation and migration in a time- and concentration-dependent manner. Moreover, we indicated that phosphatase and tension homolog (PTEN) was downregulated in myofibroblasts *in vitro* and in the contracture capsule tissues of patients *in vivo*. Additionally, western blot analysis revealed a negative correlation between the expression levels of PTEN and the fibrosis marker protein alpha smooth muscle cell actin. Methylation-specific PCR results suggested that curcumin was able to demethylate PTEN in a similar manner to the demethylation agent 5-azacytidine, increasing PTEN expression and further inhibiting phosphoinositide 3-kinase/protein kinase *B*/mammalian target of rapamycin signaling. In conclusion, our data illustrate part of the mechanism of curcumin inhibition in joint contracture. These results support the hypothesis that curcumin may potentially be used as a novel candidate for the treatment of joint contracture.

## 1. Introduction

Joint contracture is a clinical problem that affects joint function, and patients with joint contracture have a poor quality of life [1]. Fibrosis is a pathological healing process in response to chronic injury [2]. It is characterized by excessive accumulation of the extracellular matrix (ECM), which affects the architecture of the joint and ultimately results in joint stiffness [3]. Dysregulated joint contracture leads to irretrievable loss of joint function; however, there is currently no clinically advantageous drug for the prevention or treatment of joint contracture [4].

Phosphatase and tension homolog (PTEN) has attracted much attention as an antitumor gene for the treatment of various cancers, including cervical cancer [5]. It is widely acknowledged that PTEN plays a significant role in the regulation of numerous cellular processes, including proliferation, migration, and apoptosis [6]. Recently, there has been an increasing interest in the role of PTEN in the adjustment to organ fibrosis of the liver, kidney, and pulmonary system. In addition, it has been reported that aberrant methylation of the PTEN promoter can suppress PTEN expression and promote fibrosis [7–9]. However, thus far, the association between PTEN expression and joint contracture is yet to be elucidated.

Curcumin is a natural compound extracted from the root of the *Curcuma longa* plant. Various pharmacological properties of curcumin have been reported, including its antioxidant, antibacterial, anti-inflammatory, and antifibrotic characteristics [10]. Additionally, there is accumulating evidence that changes in DNA methylation play an important role in curcumin's multiple pharmacological properties and that these epigenetic events are associated with gene silencing [11]. The phosphoinositide 3-kinase (PI3K)/protein kinase *B* (Akt)/mammalian target of rapamycin (mTOR) pathway has been reported to be involved in the mechanistic actions of curcumin. To the best of our knowledge, previous studies have only indicated that curcumin has antifibrotic effects in the liver and lung and in cases of cystic fibrosis [12]. However, the mechanism and role of DNA methylation in joint contracture remains unknown.

Therefore, the current study involves an investigation of the effects of curcumin in joint contracture and an exploration of the molecular mechanisms that underlie the initiation and progression of this pathology. We hypothesized that PTEN may play an important role in the pathology of joint contracture and that curcumin may inhibit myofibroblast proliferation and migration through PTEN demethylation.

## 2. Materials and Methods

### 2.1. Ethical Statement

This study was approved by the Ethics Committee of the Third Affiliated Hospital of Sun Yat-sen University, and written informed consent was obtained from all participants prior to tissue collection. Contractive joint capsule tissue samples and normal capsule tissue samples were obtained from patients who underwent surgery at the Department of Joint Surgery and Trauma, the Third Affiliated Hospital of Sun Yat-sen University, between January 2015 and July 2017. All tissues were immediately frozen in liquid nitrogen and stored at −80°C until processing.

### 2.2. Reagents and Drug Compounds

Curcumin (1,7-bis(4-hydroxy-3-methoxyphenyl)-1,6-heptadiene-3,5-dione) was purchased from Sigma-Aldrich (Merck KGaA, Darmstadt, Germany). Stock solutions of curcumin were prepared in dimethyl sulfoxide (DMSO; Sigma-Aldrich). The primary and secondary antibodies used were as follows: KL rabbit anti-human monoclonal (1 : 1,000; ab181373; Abcam, Cambridge, MA, USA), DNA methyltransferase 1 rabbit anti-human monoclonal (1 : 2,000; #5032; Cell Signaling Technology, Inc., Danvers, MA, USA), and horseradish peroxidase-conjugated anti-rabbit IgG (1 : 3,000; #7074; Cell Signaling Technology). TRIzol® reagent was purchased from Invitrogen (Thermo Fisher Scientific, Inc., Waltham, MA, USA), and 5-aza-2-deoxycytidine (5-aza) was purchased from Sigma-Aldrich (Merck KGaA) [13].

### 2.3. Isolation and Identification of Fibroblasts

Joint capsules were collected from patients with or without joint contracture and separated into the contracture group (with contracture) and control group (without contracture). All patients were informed of the purpose and procedure of the study and agreed to tissue donation; written informed consent was obtained from all participants. The capsule samples were used for the isolation and culture of fibroblasts. Capsules were cut into 1 mm × 1 mm sections and placed in Dulbecco's Modified Eagle's Medium (DMEM; Nanjing KeyGen Biotech Co., Ltd., Nanjing, China). After 1 week, fibroblasts that had migrated from the capsule tissues were collected and identified using immunohistochemical staining.

### 2.4. Cell Lines, Cell Culture, and Treatment

Myofibroblasts were cultured in DMEM supplemented with 10% fetal bovine serum (FBS) in a 37°C incubator (5% CO_2_). Fibroblasts were induced using transforming growth factor (TGF)-*β*1 (PeproTech, Inc., Rocky Hill, NJ, USA) at a concentration of 5 ng/ml for 72 h, followed by a 24 h serum starvation period [14]. Curcumin was dissolved in DMSO (Nanjing KeyGen Biotech) to a stock concentration of 30 mM and diluted in DMEM to a final concentration of 30 *μ*M. Curcumin (30 *μ*M) was added to the myofibroblasts for a further 72 h. All control experiments included 0.1% DMSO, which is not reported to affect cell viability [15]. For DNA demethylation, cells were treated with 5 *μ*M 5-aza in DMEM (10% FBS) for 72 h. The doses were based on previous reports [16, 17]. Cells were harvested for protein extraction and DNA and RNA isolation, respectively.

### 2.5. Cell Viability Assay

Cell proliferation potential was determined using the Cell Counting Kit-8 (CCK-8) assay (Nanjing KeyGen Biotech) according to the manufacturer's instructions. Myofibroblasts were seeded into 96-well plates (Corning Inc., Corning, NY, USA) at a density of 1 × 10^3^ cells/well and treated with different concentrations of curcumin. Cell viability (%) = (*A*−*C*)/(*B*−*C*), where *A* is the absorbance of the experimental well, *B* is the mean absorbance of control wells without treatment, and *C* is the mean absorbance of blank wells.

### 2.6. Cell Migration Assay

Cell migration was analyzed using a Transwell assay. Following curcumin treatment for 24 h, 1 × 10^4^ fibroblasts were seeded into the upper chamber of a Transwell plate (Corning Inc.) in serum-free medium, while 600 *µ*l complete medium supplemented with 10% FBS was added to the lower chamber as a chemoattractant. Following incubation for 48 h at 37°C (5% CO_2_), fibroblasts on the upper surface of the chamber were removed, and migrated fibroblasts were fixed with 4% paraformaldehyde (Nanjing KeyGen Biotech) and stained with 1% crystal violet (Oneshine, Guangzhou, China). Fibroblasts were counted in five randomly selected visual fields using a microscope (Nikon Corporation, Tokyo, Japan) at ×100 magnification.

### 2.7. Collagen Assay

Fibroblasts of different groups were cultured in a growth medium supplemented with 50 mM of ascorbic acid (Wako Pure Chemical Industries, Japan) for 7 days. After removal of the medium, the extracellular collagen produced by the fibroblasts was extracted by incubating in 1 mL of pepsin solution (0.1 mg/mL in 0.5 M acetic acid) overnight at 4°C. The extracted collagen was quantized by a Sircol™ Collagen Assay kit (Biocolor, UK) as per our previous research [18].

### 2.8. Reverse Transcription-Quantitative Polymerase Chain Reaction (RT-qPCR)

RT-qPCR analysis was performed to detect PTEN and alpha smooth muscle cell actin (*α*-SMA) mRNA expression levels. Total RNA was extracted from cells using 1 ml TRIzol® (Nanjing, KeyGen Biotech) for 5 min. Subsequently, 200 *μ*l chloroform (Oneshine) was added, and the aqueous phase was collected following centrifugation at 12000 × g for 15 min at 4°C. Isopropanol (300 *μ*l; Oneshine) was added, and the mixture was centrifuged at 12000 × g for 10 min at 4°C. The RNA was resuspended in DEPC water (Nanjing KeyGen Biotech, Jiangsu) following centrifugation at 7500 × g for 5 min at 4°C. PCR reactions were conducted on the ABI 7500-Fast Real-Time PCR system (Applied Biosystems; Thermo Fisher Scientific Inc.) using the One Step SYBR® PrimeScript™ RT-PCR kit II (Takara Biotechnology Co., Ltd., Dalian, China). Thermocycling conditions were as follows: 95°C for 2 min, followed by 40 cycles of 95°C for 15 s, 60°C for 30 s, and 72°C for 30 s. The PCR primers are displayed in Table 1. *β*-actin was employed as an endogenous control.

### 2.9. Protein Extraction and Western Blot Analysis

For western blot analysis, treated cells were harvested at the indicated times by the addition of ice-cold lysis buffer (Nanjing KeyGen Biotech, Jiangsu) for 15 min. The homogenate was centrifuged at 12,000 g for 10 min at 4°C. Equal amounts of protein from cell lysates were separated by SDS-PAGE and transferred to polyvinylidene difluoride membranes (Millipore, Billerica, MA, USA). After being blocked overnight in 5% nonfat milk, the membranes were incubated at 4°C with the following primary antibodies: anti-*α*-SMA, anti-Akt, anti-phosphor-Akt, anti-PTEN, anti-phosphor-PTEN, anti-p-mTOR, and anti-mTOR (Cell Signaling Technology, Inc., Danvers, MA, USA). The membranes were washed three times and incubated with a horseradish peroxidase-conjugated secondary antibody (Abcam, Cambridge, UK) for 1 h at 37°C. Band densities were analyzed using Image-Pro Plus software 6.0, and relative protein expression was represented as the density ratio of the samples vs. that of *β*-actin.

### 2.10. Histology/Immunohistochemical Staining

Briefly, paraffin sections were stained with picrosirius red or Masson's trichrome (Sigma; Merck KGaA) according to standard procedures. Images were captured using an IX-70 microscope (Olympus Corporation, Tokyo, Japan). The collected tissues were promptly fixed with 4% paraformaldehyde in PBS (pH 7.4), dehydrated in graded ethanol, and embedded in paraffin. The slides were deparaffinized with hydration and heated at 95°C in 0.01 M sodium citrate buffer (pH 6.0) for antigen retrieval. The slides were incubated in 3% hydrogen peroxide solution and 5% goat serum and subsequently rinsed in PBS. A solution containing polyclonal antibodies to PTEN was dropped on the slides and incubated at 4°C overnight. The slides were then rinsed with PBS and incubated with a second antibody labeled with streptavidin-biotin-peroxidase. After being rinsed in PBS, the slides were incubated with 3,3-diaminobenzidine-tetrahydrochloride, counterstained with Mayer's haematoxylin, and observed using a fluorescence microscope [19].

### 2.11. Cell Transfection

Myofibroblasts were seeded into 24-well plates (Corning Inc.) at a density of 1.5 × 10^5^ cells/well. The cells were transfected with 50 nM PTEN siRNA or negative control siRNA for 48 h using a predesigned siRNA kit (Guangzhou RiboBio Co., Ltd., Guangzhou, China) according to the manufacturer's instruction.

### 2.12. Methylation-Specific PCR (MSP) Analysis

Genomic DNA isolated from fibroblasts was treated with bisulfate using the EZ DNA Methylation Gold Kit (Zymo Research Corp., Irvine, CA, USA) according to the manufacturer's protocol. The modified DNA was amplified using the following primers: PTEN-M forward, 5′-AGTTTTTATTTTTAGGGTAAACGAGTC-3′, and reverse, 5′-AACCTACTATTATATCGCCAACGTA-3′; PTEN-U forward, 5′-TTTTTATTTTTAGGGTAAATGAGTTGA-3′, and reverse, 5′-AAACAACCTACTATTATATCACCAACAT-3′. According to the manufacturer's protocol, MSP reactions were conducted using the QuantiTect® SYBR® Green PCR kit (Qiagen, China Co., Ltd., Shanghai, China). All experiments were performed at least 3 times.

### 2.13. Statistical Analysis

Data were represented as the mean ± standard error of the mean, in three separate experiments performed in triplicate. The Student's *t*-test was conducted when comparing 2 groups, and one-way analysis of variance was conducted when comparing >2 groups, using GraphPad Prism 7.0 software (GraphPad Software, Inc., La Jolla, CA, USA). *P* < 0.05 was considered to indicate a statistically significant difference.

## 3. Results

### 3.1. Curcumin Inhibits the Proliferation and Migration of Myofibroblasts

#### 3.1.1. Identification of Myofibroblasts and *α*-SMA Expression

**Figure 1:**
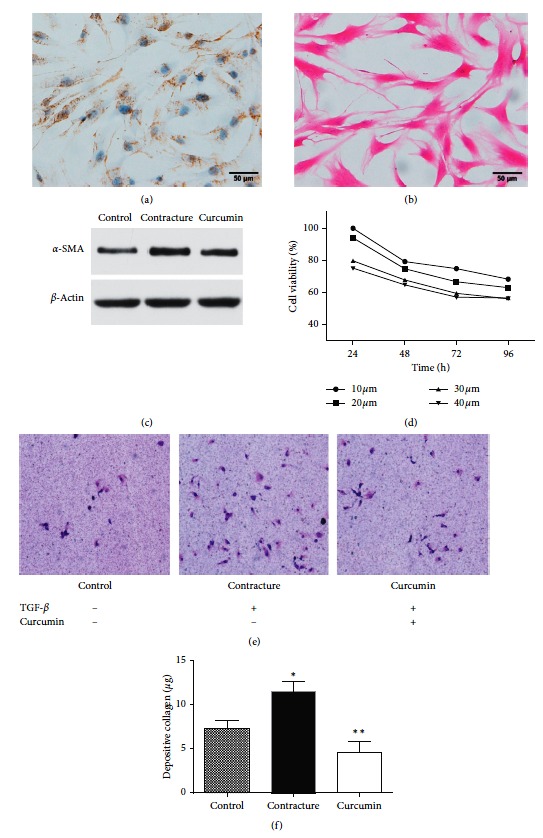
Curcumin inhibits the proliferation and migration of myofibroblasts. (a, b) Immunohistochemical and haematoxylin-eosin staining to demonstrate the morphological configuration of the myofibroblast. (c) Western blot analysis revealed that in the contracture group, the fibrosis marker *α*-SMA expression was higher compared with the normal group and that the *α*-SMA expression level was decreased following treatment with curcumin. (d) Curcumin inhibited the proliferation of myofibroblasts, as determined by the results of the Cell Counting Kit-8 assay. (e) Transwell assay showed the myofibroblast migration ability in different patient groups (magnification, ×40). *α*-SMA, alpha smooth muscle cell actin. (f) Collagen assays revealed the collagen expression in the contracture group was elevated compared with the control group, while it was decreased in the curcumin group. ^*∗*^*P* < 0.05 relative to the control group. ^*∗∗*^*P* < 0.05 relative to the contracture group.

Immunohistochemical staining showed that there were vimentin (+) particles in the cytoplasm. Haematoxylin-eosin staining showed that the fibroblasts were fusiform and bunchy. Both immunohistochemical and HE staining confirmed that the primary cells isolated from the joint capsule were myofibroblasts (Figures 1(a) and 1(b)). The expression of *α*-SMA was also determined, and the expression level served as a marker for the severity of arthrofibrosis [20]. *α*-SMA expression in the contracture group was higher than that in the normal group. Following treatment with curcumin, the expression level of *α*-SMA protein was attenuated (Figure 1(c)), indicating that curcumin can suppress the proliferation of myofibroblasts.

#### 3.1.2. Curcumin Inhibits Myofibroblast Proliferation

Previous studies have reported that curcumin inhibits fibrosis in a time- and dose-dependent manner. To investigate the appropriate curcumin concentration for use in myofibroblasts, the toxicity of curcumin at concentrations between 10 and 40 *μ*M for different time periods (24, 48, 72, and 96 h) was assessed using the CCK-8 assay [11, 22]. An increase in curcumin concentration between 10 and 30 *μ*M progressively inhibited myofibroblast proliferation, whilst curcumin concentrations between 30 and 40 *μ*M had no obvious effect on myofibroblast proliferation (Figure 1(d)). These results suggested that curcumin was able to inhibit the growth of myofibroblasts in a concentration- and time-dependent manner. In order to reduce drug toxicity, we selected 30 *μ*M as an appropriate drug concentration for subsequent studies to explore the underling molecular mechanisms of curcumin.

#### 3.1.3. Curcumin Inhibits Myofibroblast Migration

Transwell assay results showed that there were more myofibroblasts in the contracture group compared with the control group. Following curcumin treatment, the number of myofibroblasts decreased. Transwell assay revealed that the migrational ability of myofibroblasts was decreased in the curcumin group, compared with the contracture group (Figure 1(e)).

#### 3.1.4. Curcumin Inhibits the Collagen Expression of the Fibroblasts

Collagen assay revealed the collagen expression in the contracture group was elevated compared with the control group, while it was decreased in the curcumin group (Figure 1(f)).

### 3.2. PTEN Is Downregulated and p-PTEN Is Upregulated during Fibrosis

**Figure 2:**
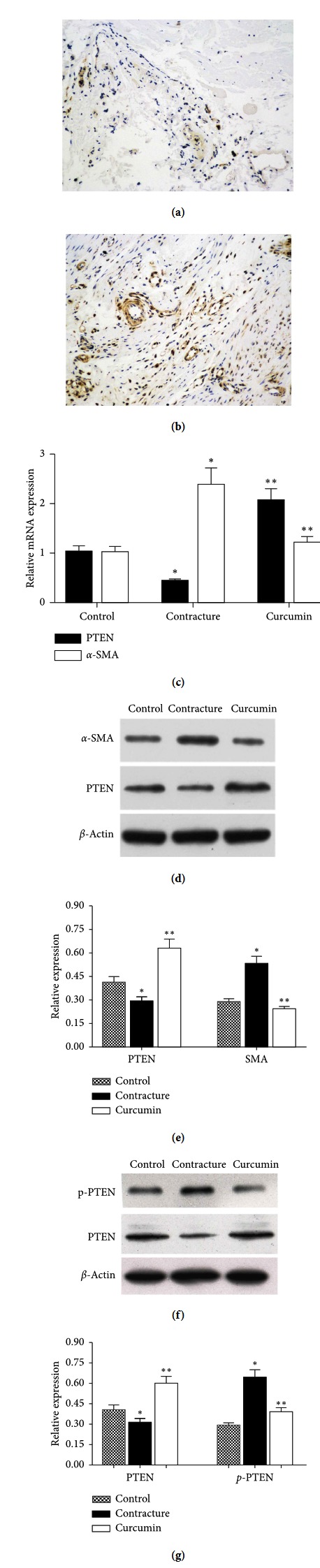
PTEN expression levels *in vivo*, *in vitro*, and in the curcumin group. (a, b) Immunohistochemical comparison of the control and the contracture group tissues (magnification, ×200). (c) Polymerase chain reaction revealed that PTEN expression levels in the contracture group were decreased compared with those in the control group, whilst they were elevated in the curcumin group. (d, e) Western blot analysis revealed that PTEN expression in the contracture group was decreased compared with the control group, while it was elevated in the curcumin group. ^*∗*^*P* < 0.05 relative to the control group. (f, g) Western blot analysis revealed that PTEN expression in the contracture group was decreased compared with the control group, while it was elevated in the curcumin group. p-PTEN expression in the contracture group was elevated compared with the control group, while it was decreased in the curcumin group. ^*∗*^*P* < 0.05 relative to the control group. ^*∗∗*^*P* < 0.05 relative to contracture group. PTEN, phosphatase and tension homolog; p-PTEN, phosphorylation of phosphatase and tension homolog.

Fibrosis tissue samples were assessed using immunohistochemistry. PTEN expression was suppressed in fibrotic capsular tissues compared with normal capsule tissues (Figures 2(a) and 2(b)). PTEN RNA and protein in the contracture group were reduced; p-PTEN protein was increased compared with the control group (*P* < 0.05). Interestingly, following the addition of curcumin, the expression levels of PTEN RNA and protein in fibroblasts increased once more; p-PTEN protein was downregulated, compared with those of the contracture group (Figures 2(c)–2(g)) (*P* < 0.05).

### 3.3. PTEN Exhibits Antifibrotic Functions in Myofibroblasts

**Figure 3:**
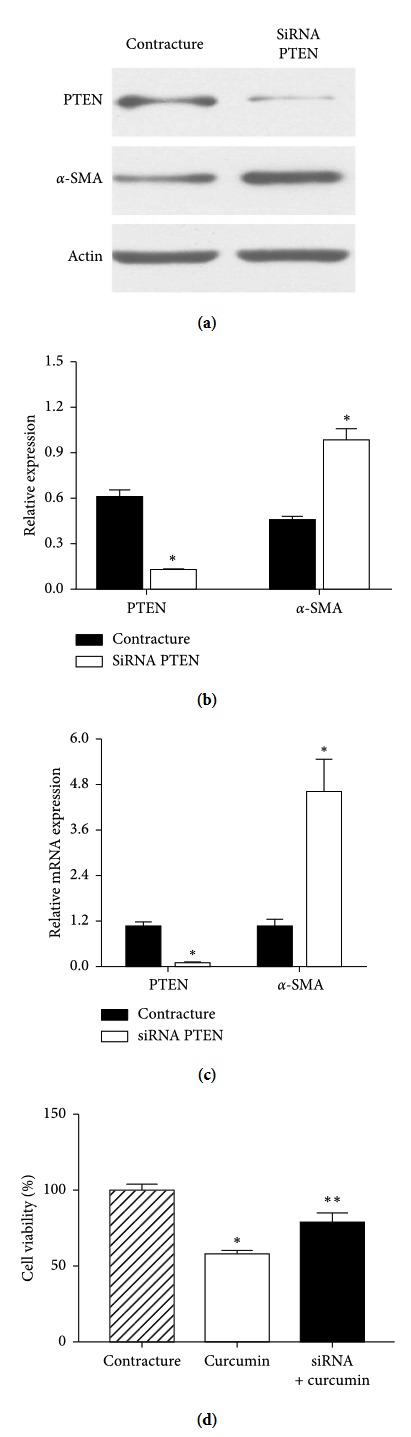
PTEN exhibits antifibrotic effects on myofibroblasts. (a, b) Following siRNA knockdown, western blot analysis revealed a decrease in the expression of PTEN, while that of the fibrosis marker protein *α*-SMA increased, compared with the contracture group. (c) Polymerase chain reaction revealed decreased PTEN expression levels in the siRNA PTEN knockdown group, while *α*-SMA expression levels were increased, compared with the contracture group. (d) Cell Counting Kit-8 analysis showed that myofibroblast proliferation was suppressed in the curcumin group, while in the siRNA PTEN + curcumin group, proliferative ability was increased, compared with the curcumin group. ^*∗*^*P* < 0.05 relative to the contracture group. ^*∗∗*^*P* < 0.05 relative to the curcumin group. PTEN, phosphatase and tension homolog; *α*-SMA, alpha smooth muscle cell actin; siRNA, small-interfering RNA.

Following the siRNA knockdown of PTEN, *α*-SMA RNA and protein expression levels increased compared with the contracture group, as determined by RT-qPCR and western blot analysis (Figures 3(a)–3(c)) (*P* < 0.05). CCK-8 analysis showed that myofibroblast proliferation was inhibited in the curcumin group, while in the siRNA PTEN + curcumin coculture group, this effect was reversed (*P* < 0.05). This implied that PTEN may possess an antifibrotic function in myofibroblasts and that PTEN knockdown may attenuate the antifibrotic effects induced by curcumin.

### 3.4. Methylation Inhibits PTEN Gene Expression and Curcumin Is Able to Demethylate PTEN

**Figure 4:**
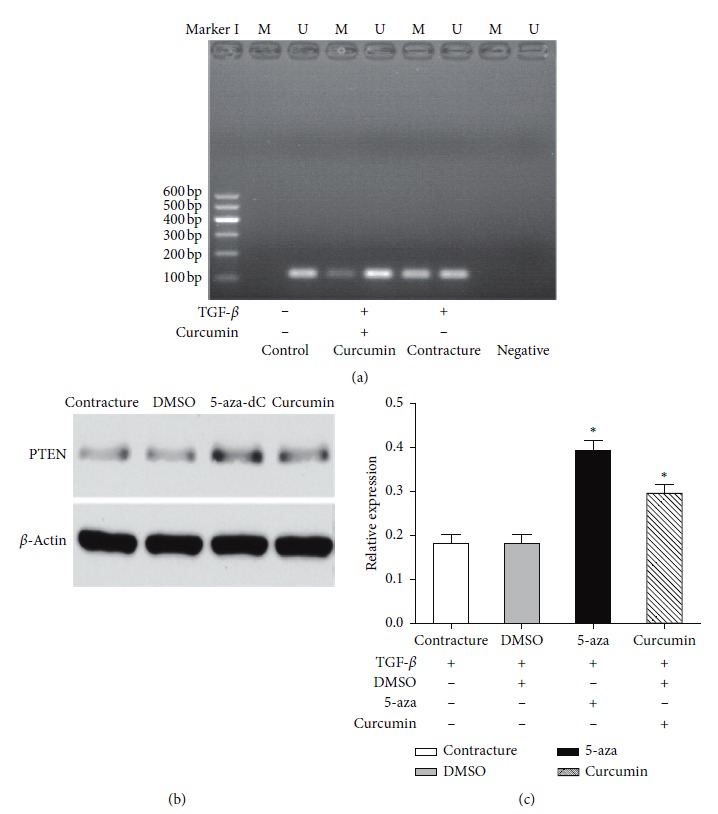
Methylation inhibits PTEN gene expression, and curcumin can demethylate PTEN. (a) Methylation-specific polymerase chain reaction assay revealed no methylated bands in the control group. Methylated bands were detected in the transforming growth factor-*β*-induced contracture group, while in the curcumin group, the methylated bands were detected to a lesser degree. (b, c) Western blot analysis showed that both the 5-azacytidine group and the curcumin group exhibited raised PTEN expression levels in myofibroblasts, compared with the contracture and DMSO groups. ^*∗*^*P* < 0.05 relative to the contracture group. PTEN, phosphatase and tension homolog.

MSP-PCR revealed that PTEN DNA was methylated to a greater degree in the contracture group compared with that in the control group, whilst in the curcumin group, the degree of methylation was reduced (Figure 4(a)). Western blot analysis revealed that the methylation of PTEN in the contracture group resulted in a reduction in PTEN expression in myofibroblasts. PTEN expression in the DMSO group was similar to that in the contracture group, and the differences between these two groups were not statistically significant. However, following treatment with 5-aza, PTEN was able to be demethylated, and western blot analysis revealed that PTEN expression was upregulated compared with that in the contracture group. Moreover, curcumin enhanced PTEN expression to the same degree as the demethylation agent 5-aza (Figures 4(b) and 4(c)).

### 3.5. Curcumin Suppresses Fibrosis by Upregulating PTEN Expression via the PI3K/Akt/mTOR Signaling Pathway

**Figure 5:**
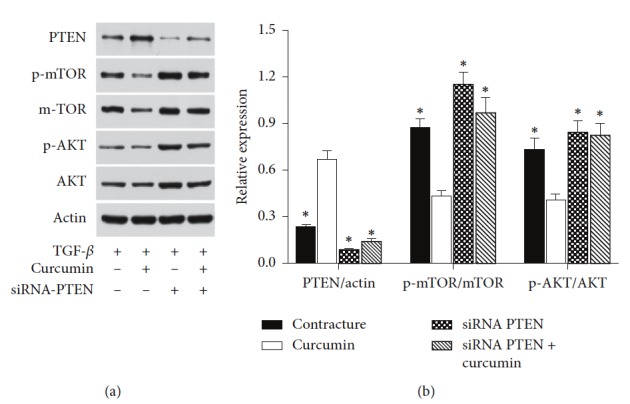
Curcumin suppresses fibrosis partly by downregulating the expression of PTEN via the PI3K/Akt/mTOR signaling pathway. (a) Western blot analysis was used to determine the protein levels of PTEN, p-mTOR, mTOR, p-AKT, and AKT. The corresponding internal control was actin. (b) The ratios of PTEN/actin, p-mTOR/TOR, and p-Akt/Akt in the contracture group, the curcumin group, the siRNA PTEN group, and the siRNA PTEN + curcumin group were calculated. ^*∗*^*P* < 0.05 relative to the curcumin group. PTEN, phosphatase and tension homolog; PI3K/Akt/mTOR, phosphoinositide 3-kinase/protein kinase *B*/mammalian target of rapamycin; p-AKT, phosphorylated AKT; p-mTOR, phosphorylated mTOR; siRNA, small-interfering RNA.

To explore whether curcumin regulates PTEN expression via the PI3K/Akt/mTOR pathway, protein expression levels of PTEN, Akt, p-Akt, mTOR, and p-mTOR were assessed in myofibroblasts, using western blot analysis. As shown in Figures 5(a) and 5(b), the ratios of PTEN/actin, p-Akt/Akt, and p-mTOR/mTOR were markedly reduced in the curcumin-treated group compared with the contracture group (*P* < 0.05). This indicated that PTEN participates in the PI3K/Akt/mTOR signaling pathway and that in myofibroblasts, the phosphorylation levels of Akt and mTOR were attenuated by curcumin treatment. These findings indicated that PI3K/Akt/mTOR signaling can be inhibited by curcumin.

Interestingly, siRNA knockdown of PTEN restored the ratios of p-Akt/Akt and p-mTOR/TOR once more. At this point, even additional curcumin treatment of the siRNA PTEN + curcumin group did not decrease the ratios of p-Akt/Akt and p-mTOR/TOR (Figures 5(a) and 5(b)). This implied that curcumin exhibited its inhibition of PI3K/AKT/mTOR signaling by regulating PTEN expression. This partially elucidates the molecular mechanisms by which curcumin exerts its antifibrotic effect.

## 4. Discussion

Joint contracture remains a challenging clinical problem in the medical arena. Up until now, the most common treatments for arthrofibrosis have been passive rehabilitation techniques such as stretching. If conservative treatment does not result in a favorable outcome, surgical intervention may be used to improve the range of joint motion. There is still no identified effective treatment for the prevention of joint contracture. Myofibroblast activation can cause excessive deposition of the ECM in the posterior joint capsule, and the expression of profibrogenic genes, such as collagen type I and *α*-SMA, subsequently increases [21–25]. Collectively, these pathological changes lead to a restricted range of motion. Due to the articular structure, contracture is irreversible, thus preventing the progression of arthrofibrosis is more important in the early phases than when joint contracture has already occurred. In the present study, curcumin significantly inhibited the migration and proliferation of myofibroblasts. Its application may therefore be beneficial in the early phases of arthrofibrosis. To the best of our knowledge, there have been no previous reports on the effects and potential molecular mechanisms of curcumin in joint contracture.

Curcumin is a polyphenol pigment extracted from turmeric. It has been reported to exhibit extensive pharmacological activity, with anti-inflammatory, antioxidative, and antitumor properties. In recent years, several studies have investigated the antifibrotic effects of curcumin on organs such as the liver and kidney [11, 26–29]. However, thus far, no accounts of curcumin use for joint contracture have been reported. Our data confirmed the antifibrotic effects of curcumin in joint contracture. Both CCK-8 and Transwell assays demonstrated that curcumin can significantly inhibit the proliferation and migration of myofibroblasts in a dose-independent manner (Figures 1(d) and 1(f)). Collagen is the major constituent in fibrous tissue, and the development of joint contracture has a close relationship with the deposition of fibroblast collagen [30]. The result of collagen assay indicated that curcumin could inhibit the expression of collagen synthesis by fibroblasts in an *in vitro* fibrous model (Figure 1(f)).

The present study also implicated that curcumin may be used for the treatment of joint contracture, as no effective medicine currently exists for the treatment of the condition.

In the current study, it was observed that PTEN, a recognized tumor suppressor gene, plays an important role in the regulation of myofibroblast proliferation. Its expression is decreased in a number of different malignancies, including colorectal, breast, ovarian, and prostate cancer [5, 6]. Likewise, reports have also revealed that PTEN expression is associated with renal and liver fibrosis [7, 31]. However, there are a limited number of investigations into PTEN expression in the joint capsule. Our research verified that PTEN expression was lower in contracture capsule tissues compared with normal capsule tissues, as determined by immunohistochemistry, RT-qPCR, and western blot analysis (Figure 2). Myofibroblasts play a crucial role in joint contracture and are characterized by the expression of *α*-SMA. PTEN expression was inversely correlated with *α*-SMA expression in the contracture group, compared with that in the control group. Furthermore, we also found the protein expression of p-PTEN in the contracture group was elevated compared with the control group, while it was decreased in the curcumin group (Figures 2(f) and 2(g)). Curcumin can upregulate PTEN expression and simultaneously inhibite p-PTEN expression and the proliferation and migration of myofibroblasts.

Following PTEN knockdown with siRNA, the inhibitory effects of curcumin were reversed (Figure 3). The results of the CCK-8 assay revealed that PTEN siRNA can attenuate the inhibitory effects of curcumin on the proliferation of myofibroblasts. It also implicated that PTEN protein may regulate the proliferation of myofibroblasts. In the absence of PTEN, the suppressed proliferation effect of curcumin was attenuated (Figure 3). PTEN is able to regulate the expression of collagen, which is resistant to joint contracture. Therefore, the upregulation of PTEN may be a part of the mechanism by which curcumin inhibits myofibroblast proliferation.

More recent studies have confirmed that epigenetic regulatory processes, including methylation, can regulate gene expression and influence the development of fibrosis [16, 32]. To further understand the epigenetic regulation of PTEN in joint contracture, MSP was carried out. The results of MSP revealed that PTEN was methylated in the contracture group compared with the control group yet demethylated in the curcumin group (Figure 4(a)). Concurrently, western blot analysis showed that aberrant PTEN methylation in the contracture group resulted in the downregulation of PTEN expression. However, PTEN expression was restored in the 5-aza group and the curcumin groups (Figures 4(b) and 4(c)). DMSO is a commonly used solvent in curcumin and 5-aza preparations and a frequently used demethylation agent. The results of western blotting implied that curcumin had a similar effect on the demethylation of PTEN and the upregulation of PTEN expression as the DNA methyltransferase inhibitor 5-aza.

The PI3K/Akt/mTOR signaling pathway plays an important role in a number of cellular processes, including cell growth, migration, and differentiation. Aberrant activation of the PI3K/Akt/mTOR pathway has been associated with cell proliferation, which is often observed in different tumor types. Inhibiting the PI3K/Akt/mTOR signaling pathway is a useful way to suppress proliferation, which is the mechanism of action for various antitumor agents [33–35].

**Figure 6:**
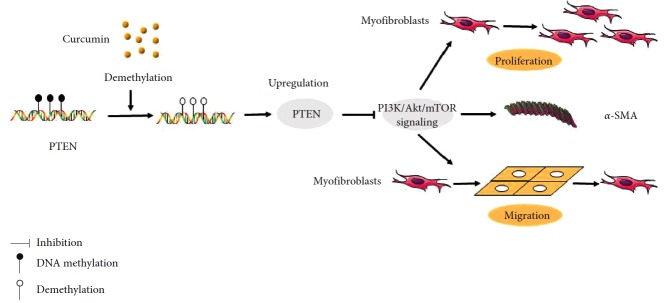
Myofibroblasts were treated with curcumin. Curcumin restored PTEN expression, downregulated *α*-SMA expression, and suppressed the proliferation and migration of myofibroblasts by demethylation and inhibition of PI3K/Akt/mTOR signaling.

Based on western blot analyses, we observed that PTEN expression was elevated with the suppression of Akt and mTOR phosphorylation in the curcumin group. Additionally, following PTEN siRNA knockdown, the expression of p-Akt and p-mTOR was increased (Figure 5). The inhibitory effects of curcumin on the phosphorylation of Akt and mTOR may also be attenuated. Based on the above results, the present study proposed that curcumin was able to demethylate PTEN and upregulate its expression, thus inhibiting the PI3K/Akt/mTOR signaling pathway. This has elucidated part of the molecular mechanism by which curcumin exerts its antifibrotic effect, which may be a novel target for the treatment of joint contracture.  Figure 6 shows the mechanism how curcumin regulated the myofibroblast.

According to the aforementioned results, PTEN is a novel regulator of myofibroblast activation in joint contracture. Curcumin is able to retard the progression of joint contracture through the upregulation of PTEN, by demethylation and inhibition of elements of the PI3K/Akt/mTOR pathway. Additional research is required to characterize the CpG promoter methylation sites, in addition to further *in vivo* experimentation. This study offers a novel and promising strategy to prevent the development of joint contracture, where curcumin may be used to treat the condition.

## Figures and Tables

**Table 1:** Primers used for reverse transcription-quantitative polymerase chain reaction analysis.

Name	Primer sequence (5′–3′)	Product size (bp)
PTEN	Forward: 5′-TGGATTCGACTTAGACTTGACCT-3′	231
	Reverse: 5′-GGTGGGTTATGGTCTTCAAAAGG-3′	

*α*-SMA	Forward: 5′-ACGAGACCACCTACAACAGCAT-3′	269
	Reverse: 5′-CTCGTCGTACTCCTGCTTGGT-3′	

*β*-Actin	Forward: 5′-AACAGTCCGCCTAGAAGGAC-3′	281
	Reverse: 5′-CGTTGACTACCGTAAAGACC-3′	

PTEN: PTEN, phosphatase and tension homolog; *α*-SMA: *α*-smooth muscle actin.

## Data Availability

The data used to support the findings of this study are available from the corresponding author upon request.
